# Valence band offset of InN/BaTiO_3 _heterojunction measured by X-ray photoelectron spectroscopy

**DOI:** 10.1186/1556-276X-6-316

**Published:** 2011-04-08

**Authors:** Caihong Jia, Yonghai Chen, Yan Guo, Xianglin Liu, Shaoyan Yang, Weifeng Zhang, Zhanguo Wang

**Affiliations:** 1 Key Laboratory of Semiconductor Material Science, Institute of Semiconductors, Chinese Academy of Science, P.O. Box 912, Beijing 100083, PR China; 2 Key Laboratory of Photovoltaic Materials of Henan Province and School of Physics Electronics, Henan University, Kaifeng 475004, PR China

## Abstract

X-ray photoelectron spectroscopy has been used to measure the valence band offset of the InN/BaTiO_3 _heterojunction. It is found that a type-I band alignment forms at the interface. The valence band offset (VBO) and conduction band offset (CBO) are determined to be 2.25 ± 0.09 and 0.15 ± 0.09 eV, respectively. The experimental VBO data is well consistent with the value that comes from transitivity rule. The accurate determination of VBO and CBO is important for use of semiconductor/ferrroelectric heterojunction multifunctional devices.

## Introduction

The semiconductor-ferroelectric heterostructures have attracted much attention due to their large potential for new multifunctional electronic and optoelectronic device applications [1-5]. Hysteresis properties of the ferroelectric polarization allow for bistable interface polarization configuration The polarization coupling between the fixed permanent semiconductor dipole and the switchable ferroelectric dipole can be exploited to modify the electronic and the optical properties of a semiconductor heterostructure. Recently, GaN-based high electron mobility transistor devices have been integrated on ferroelectric LiNbO_3_, providing the compact optoelectronic/electronic chips with increased cost savings and added functionality [6]. The semiconductor-ZnO/ferroelectric-BaTiO_3 _(BTO) heterostructure metal-insulator-semiconductor field-effect transistors have been demonstrated, in which the polarization of the BTO can be used to control the free carrier concentration in the ZnO channel [7]. In order to fully exploit the advantages of semiconductor-ferroelectric heterostructures, other combinations such as InN/BTO should be explored. As a remarkable ferroelectric material with a high relative permittivity, BTO can be used as a gate dielectric for InN-based field-effect transistor. More importantly, InN/BTO heterojunction is promising for fabricating optical and electrical devices since oxidation treatment is found to reduce the surface electron accumulation of InN films [8].

For heterostructure devices, it is important to accurately determine the valence and the conduction band offsets, which dictate the degree of charge carrier separation and localization. However, up to date, there is lack of experiment data available on the interface band alignment parameters for InN/BTO heterojunction. In this letter, we determine the VBO as well as conduction band offset (CBO) values of the InN/BTO heterojunction using X-ray photoelectron spectroscopy (XPS).

## Experimental

Three samples (bulk BTO, thick InN/BTO, and thin InN/BTO) were studied in this work, namely, a bulk commercial (001) BTO substrate, a thick 200-nm InN layer and a thin 5-nm InN layer grown on the commercial (001) BTO substrates, respectively. To get a clean interface, the BTO substrate was cleaned with organic solvents and rinsed with de-ionized water sequentially before loading into the reactor. The thick and thin heterostructures of InN/BTO were deposited by metal-organic chemical vapor deposition (MOCVD) at 520°C. More growth condition details of the InN layer can be found in our previous report [9]. XPSs were performed on a PHI Quantera SXM instrument with Al K*α *(*hν *= 1486.6 eV) as the X-ray radiation source, which had been carefully calibrated on work function and Fermi energy level (*E*_F_). Because a large amount of electrons are excited and emitted from the sample, the sample is always positively charged and the electric field caused by the charge can affect the measured kinetic energy of photoelectron. Charge neutralization was performed with an electron flood gun and all XPS spectra were calibrated by the C1s peak at 284.8 eV from contamination to compensate the charge effect. Since only the relative energy position in each sample is needed to determine the VBO, the absolute energy calibration for a sample has no effect on the ultimate result. The surfaces of samples were examined initially by low-resolution survey scans to determine which elements were present. Very high-resolution spectra were acquired to determine the binding energy of core level and the valence band maximum energy in the survey spectra.

## Results and discussion

**Figure 1:**
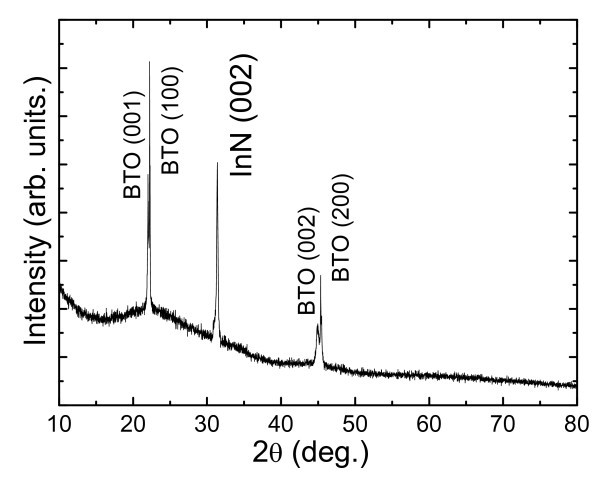
**X-ray *θ*-2*θ *diffraction pattern of the thick InN films on BTO substrates**.

In X-ray *θ*-2*θ *diffraction measurements, as shown in Figure 1, the thick InN/BTO sample presented the only peak of InN (0002) reflection and no other InN-related peaks were observed, implying a complete c-axis-oriented growth of the InN layer. The VBO (ΔE_V_) can be calculated from the formula(1)

where  is the energy difference between In3d and Ti2p core levels (CLs) measured in the thin heterojunction InN/BTO, while  and  are the valence band maximum (VBM) energies with reference to the CL positions of bulk BTO and thick InN film, respectively. Because all the samples were exposed to air, there must be some impurities (e.g., oxygen and carbon) existing in the sample surface, which may prevent the precise determination of the positions of the VBMs. To reduce the undesirable effects of surface contamination, all the samples were cleaned by Ar^+ ^bombardment at a low sputtering rate to avoid damage to the samples. After the bombardment, peaks related to impurities were greatly reduced, and no new peaks appeared.

**Figure 2:**
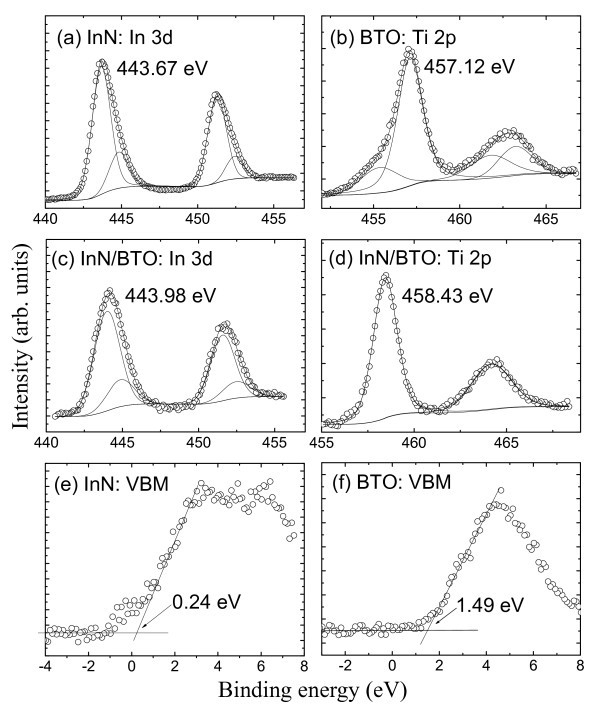
**In3d spectra recorded on InN (a) and InN/BTO (e), Ti2p spectra on BTO (c) and InN/BTO (f), and VB spectra for InN (b) and BTO (d)**. All peaks have been fitted to Voigt line shapes using Shirley background, and the VBM values are determined by linear extrapolation of the leading edge to the base line. The errors in the peak positions and VBM are ±0.03 and ±0.06 eV, respectively.

Figure 2 shows the XPS Ti2p and In3d CL narrow scans and the valence band spectra from the bulk BTO, thick InN, and thin InN/BTO samples, respectively. All the CL spectra were fitted to Voigt (mixed Lorentz-Gaussian) line shape with a Shirley background. The uncertainty of the CL position is less than 0.03 eV, evaluated by numerous fittings with different parameters. The VBM positions were determined by linear extrapolation of the leading edge of the VB spectra recorded on the bulk BTO and thick InN film to the base lines to account for the instrument resolution-induced tail [10]. Evidently, the VBM value is sensitive to the choice of points on the leading edge used to obtain the regression line [11]. Several different sets of points were selected over the linear region of the leading edge to perform regressions, and the uncertainty of VBO is found to be less than 0.06 eV in the present work.

For the In3d spectra of both the InN and the thin InN/BTO samples, additional low intensity higher-binding-energy components were required. These extra components are attributed to In-O bonding due to oxide contamination when InN is present at the surface [12], as shown in Figure 2a. In the thin InN/BTO sample shown in Figure 2c, they are attributed to In-O bonding at the InN/BTO interfaces, and/or inelastic losses to free carriers in the InN layer [13]. The CL peak attributed to In-N bonding locates at 443.67 ± 0.03 and 443.98 ± 0.03 eV for thick InN and thin InN/BTO, respectively, as shown in Figure 2a, c. From Figure 2b, it can be clearly seen that the Ti2p peak in the bulk BTO is not symmetric and consists of two components by careful Voigt fitting. The prominent one located at 457.12 ± 0.03 eV is attributed to the Ti emitters within the BTO substrate, which have six bonds to oxygen atoms. The other one shifting by ~2 eV to a lower binding energy is attributed to TiO_x _suboxides on account of the TiO-terminated BTO initial surface [14]. However, the Ti2p spectrum in the thin InN/BTO heterojunction is quite symmetric, indicating a uniform bonding state and the only peaks correspond to Ti-O bonds. It is interesting that the Ti2p peaks transform from asymmetry in bulk BTO to symmetry in the thin InN/BTO sample, as recently observed in the thin ZnO/BTO heterostructure [15]. The VBM value of bulk BTO is determined to be 1.49 ± 0.06 eV using the linear method. The Fermi energy level of an insulator is expected to be located in the middle of the forbidden energy gap, so the VBM will be one-half of the band gap of insulators [16]. For BTO, the VBM should be 1.55 eV calculated from the band gap of 3.1 eV [17], which is in good agreement with the measured value (1.49 ± 0.06 eV) in the present work. Using the same fitting methods mentioned above, the VBM value for the thick InN lms can be determined to be 0.24 eV, as shown in Figure 1e. Substituting the above values in Equation 1, the resulting VBO value is calculated to be 2.25 ± 0.09 eV.

The reliability of the measured result is analyzed by considering several possible factors that could impact the experiment results. The energy band bends downward at the surface of InN film and there is an electron accumulation layer [18], so the energy separation between VBM and Fermi level can be changed at the InN surface, which could impact the measured VBO values of the heterojunctions. However, both the CL emissions of In3d and Ti2p at the InN/BTO heterojunction are collected from the same surface (InN surface), thus, the surface band bending effects can be canceled out for the measurement of Δ*E*_CL_, as was the measurement of the band offset of the InN/AlN heterojunction by others [19,20].

Another factor which may affect the precision of the VBO value is the strain-induced piezoelectric field in the overlayer of the heterojunction [21]. There is a large lattice mismatch of about 7.1%  between the hexagonal apothem of InN and the  direction. It is comparable with that of the InN/ZnO heterojunction (7.7%), and the InN thin film of 5 nm is approximately treated as completely relaxed [10]. So the strain-induced piezoelectric field effect can be neglected in our experiment. Since the factors that can affect the ultimate result can be excluded from the measured result, the experimental obtained VBO value is somewhat reliable.

To further confirm the reliability of the experimental values, it would be useful to compare our VBO value with other results deduced by transitive property. For heterojunctions formed between all pairs of three materials (A, B, and C), Δ*E*_V_(A-C) can be deduced from the difference between Δ*E*_V_(A-B) and Δ*E*_V_(C-B) neglecting the interface effects [22]. The reported VBO values for ZnO/BTO and InN/ZnO heterojunctions are Δ*E*_V_(ZnO-BTO) = 0.48 eV [15], and Δ*E*_V_(InN-ZnO) = 1.76 eV [23], respectively. Then the Δ*E*_V_(InN-BTO) is deduced to be 2.24 eV, which is well consistent with our measured value 2.25 ± 0.09 eV. In addition, the resulting Δ*E*_V _is a large value for device applications which require strong carrier con nement, such as light emitters or heterostructure field effect transistors.

**Figure 3:**
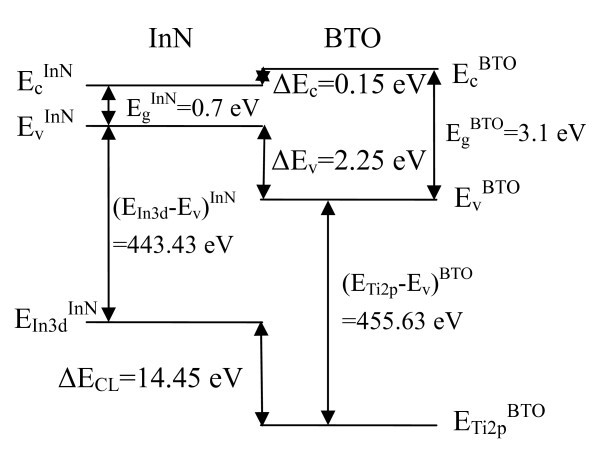
**Energy band diagram of InN/BTO heterojunction**.

Finally, the CBO (ΔE_C_) can be estimated by the formula . By substituting the band gap values at room temperature ( = 0.7 eV [23] and  = 3.1 eV [17]), Δ*E*_C _is calculated to be 0.15 ± 0.09 eV. Accordingly, a type-I band alignment forms at the heterojunction interface, as shown in Figure 3.

## Conclusions

In summary, XPS was used to measure the VBO of the InN/BTO heterojunction. A type-I band alignment with the VBO of 2.25 ± 0.09 eV and CBO of 0.15 ± 0.09 eV is obtained. The accurately determined result is important for the design and application of InN/BTO heterostructure-based devices.

## Abbreviations

CBO: conduction band offset; CLs: core levels; MOCVD: metal-organic chemical vapor deposition; VBM: valence band maximum; VBO: valence band offset; XPS: X-ray photoelectron spectroscopy.

## Competing interests

The authors declare that they have no competing interests.

## Authors' contributions

CJ carried out the experimental analysis and drafted the manuscript. YC carried out the experimental design. YG participated in the experimental analysis. XL carried out the growth and optimization of indium nitride films. SY participated in the experimental measurement. WZ participated in its design and coordination. ZW participated in the experimental design. All authors read and approved the final manuscript.
